# Multifunctional properties of bio-poly(butylene succinate) reinforced with multiwalled carbon nanotubes

**DOI:** 10.3762/bjnano.16.76

**Published:** 2025-07-03

**Authors:** Volodymyr Krasinskyi, Krzysztof Bajer, Ludmila Dulebova, Nickolas Polychronopoulos, Oksana Krasinska, Daniel Kaczor

**Affiliations:** 1 Łukasiewicz Research Network – Institute of Polymer Materials, 55 M. Skłodowska-Curie St., 87-100 Toruń, Poland; 2 Faculty of Mechanical Engineering, Technical University of Košice, Masiarska 74 St., 04001 Košice, Slovakia; 3 Department of Mechanical Engineering, University of West Attica, 250 Thivon & P. Ralli, Egaleo 12241, Athens, Greece

**Keywords:** melt compounding, multiwalled carbon nanotubes, poly(butylene succinate), structure, tribological properties

## Abstract

Recent advances in nanocomposite technology, particularly the incorporation of carbon nanotubes, have shown promise in enhancing the properties of biodegradable polymers. This study investigated the effect of a 0.5 wt % addition of multiwalled carbon nanotubes (MWCNTs) on the properties of bio-poly(butylene succinate) (BioPBS) using a masterbatch-based melt compounding method. The incorporation of MWCNTs enhanced the mechanical strength and stiffness, improved the tribological properties by reducing friction, and increased the crystallization temperature. However, it also resulted in a decrease in elasticity. Morphological analysis confirmed the uniform dispersion of the nanotubes. These findings underscore the potential of MWCNTs to tailor the properties of BioPBS for specific applications, such as in the packaging, automotive, and biomedical industries, where both biodegradability and enhanced material performance are desirable.

## Introduction

In recent years, biodegradable polymers have gained significant attention as environmentally friendly alternatives to traditional plastics. One particularly promising material is poly(butylene succinate), which exhibits a desirable combination of mechanical strength, thermal stability, and biodegradability. However, to broaden its range of applications, certain properties require enhancement, including mechanical performance, thermal and electrical conductivity, biodegradation rate, and barrier properties [1–5]. The limited biodegradability of PBS in microbial environments is primarily attributed to its high degree of crystallinity [6–8].

A well-established approach to improving polymer properties involves the incorporation of nanofillers, particularly carbon nanotubes (CNTs). Even at low concentrations, CNTs can significantly affect the morphology and properties of the polymer matrix by altering its structure, crystallinity, thermal stability, and mechanical behavior [9–11]. CNTs can be classified into two basic types, namely, single-walled carbon nanotubes (SWCNTs) and multiwalled carbon nanotubes (MWCNTs). SWCNTs exhibit slightly better electrical, mechanical, and optical properties than MWCNTs, making them more desirable for high-end applications such as nanoelectronics and advanced composites. However, the synthesis cost of SWCNTs is significantly higher, while their production yield is considerably lower than that of MWCNTs, making the latter a more economically viable option for most commercial applications. Therefore, this study focuses on investigating the effect of MWCNTs on the properties of bio-poly(butylene succinate) (BioPBS).

The effect of CNTs on various characteristics of biopolymers has been widely studied by many researchers [9–16]. Regarding PBS-based nanocomposites, most studies focus on the influence of CNTs (including modified CNTs) on their thermal behavior [17–19], crystallization [20–22], structure [14,17,21–23], and biodegradability [12]. PBS/CNT nanocomposites are primarily synthesized using two methods, that is, solution casting [18,22,24] and melt compounding [17,19,23]. In this study, the melt compounding method was chosen due to its simplicity, high throughput, and industrial feasibility.

Bandyopadhyay et al. [23] demonstrated that melt mixing 1, 2, and 3 wt % of MWCNTs functionalized with carboxyl groups with PBS in a batch mixer resulted in a uniform distribution of agglomerated MWCNTs within the polymer matrix, an increase in the storage modulus, and enhanced electrical conductivity of PBS. Moreover, MWCNTs had a significant effect on the viscoelastic properties of PBS. Wang et al. [19] reported similar results when melt mixing 0.5, 1.0, 2.0, and 3.0 wt % pristine raw MWCNTs with PBS in a mixer. Additionally, they observed an increase in the thermal stability and crystallization temperature of PBS.

Champa-Bujaico et al. [18] and Zeng et al. [22] synthesized lowly filled PBS-based nanocomposites, namely, PBSA/SWCNTs and PBS/MWCNTs, respectively, via solution casting after the pre-modification of CNTs. At an SWCNT content of 0.5 wt % in PBSA, a twofold increase in PBS stiffness was observed, along with increased crystallization and glass transition temperatures, while impact strength showed a slight decrease [18]. MWCNTs modified with poly(sodium 4-styrenesulfonate) increased the crystallization temperature of PBS by 14 °C even at a concentration of 0.05 wt % [22]. Moreover, a significant increase in electrical conductivity was observed at an MWCNT content of 0.3 wt %. These nanocomposites also exhibited a uniform MWCNTs distribution without significant agglomeration.

This study aimed to conduct a comprehensive investigation into the effect of a small addition of pure MWCNTs on the morphology, rheological, tribological, physicomechanical, and thermal properties of BioPBS (hereafter PBS). To improve the distribution of MWCNTs (hereafter CNTs) within the polymer matrix and precisely control the CNT content, a nanocomposite preparation method utilizing a polymer masterbatch was employed, involving double mixing stages in a twin-screw extruder.

## Results and Discussion

The bio(nano)composite based on PBS containing 0.5 wt % of CNTs (denoted PBS/CNT_0.5) was successfully prepared via dilution of a pre-compounded masterbatch with 10 wt % CNTs (denoted PBS/CNT_10). This specific CNT content was identified as optimal, balancing enhanced material performance with processability and cost-effectiveness. Increasing the CNT concentration beyond this level resulted in a marked rise in melt viscosity and the formation of agglomerates within the polymer matrix, which adversely affected both the processing behavior and the final properties of the composite.

**Figure 1:**
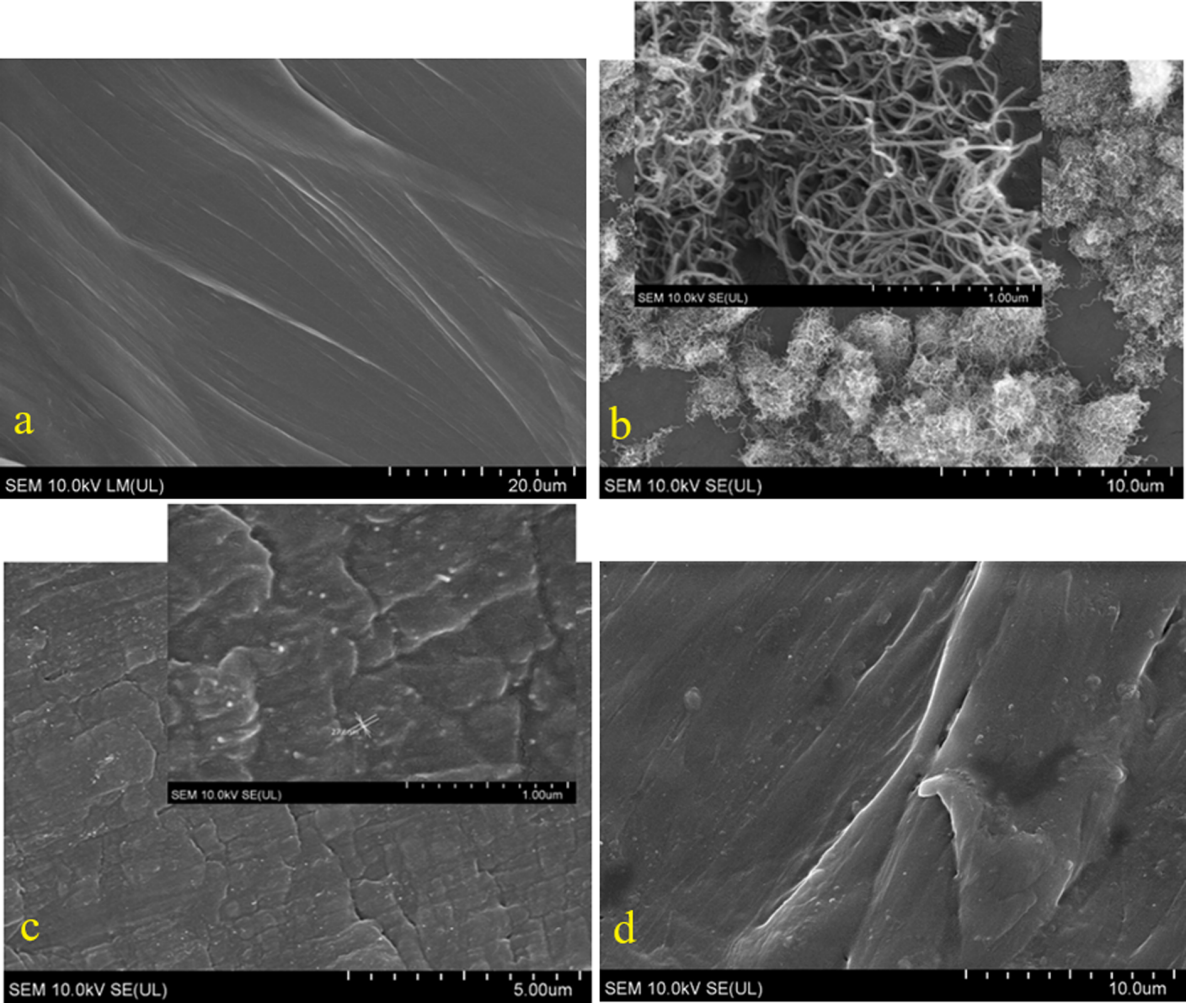
SEM images of PBS (a), CNTs (b), PBS/CNT_10 (c), and PBS/CNT_0.5 (d).

The surface morphology of the original substances and the PBS/CNTs nanocomposites was studied using SEM (Figure 1). In Figure 1a, the typical surface structure of PBS is visible. CNTs appear as agglomerates ranging from 500 nm to 200 μm, composed of woven nanotubes with an outer diameter of 14 to 28 nm and a length of up to 40 μm (Figure 1b). The microstructure of PBS/CNT_10 (Figure 1c) and PBS/CNT_0.5 (Figure 1d) samples is quite similar. In the SEM images of both samples, small white inclusions (nanotubes) ranging from 24 to 200 nm in size are evenly distributed over the surface. On the surface of the masterbatch sample (PBS/CNT_10), there are many more of these white inclusions, which is expected, as the CNT content in this sample is 20 times higher. Thus, due to the mixing of CNTs with PBS in a twin-screw extruder with a special screw configuration, the CNT agglomerates are broken down into nanosizes and uniformly distributed within the polymer matrix.

**Figure 2:**
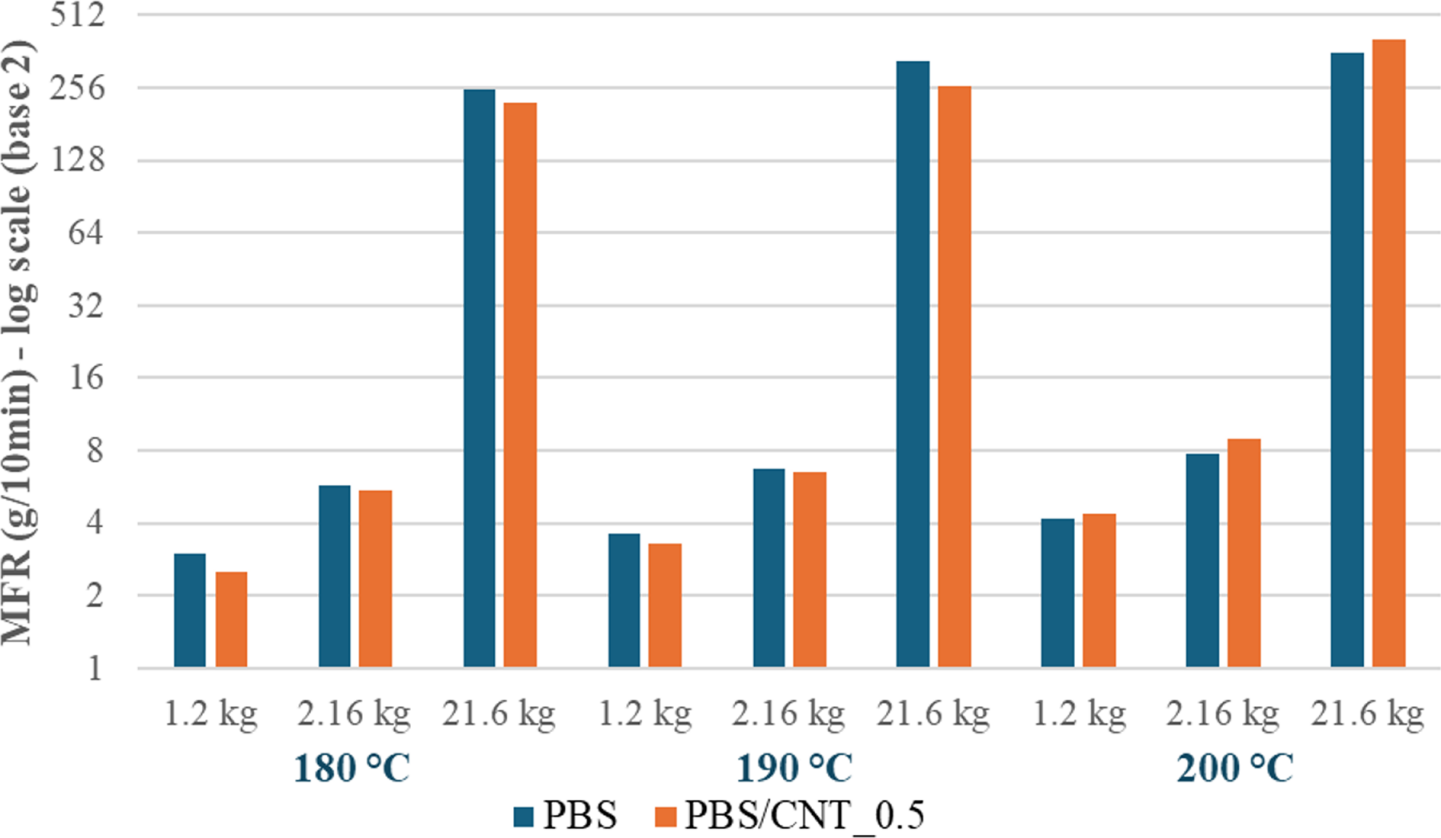
MFR of pure PBS and PBS/CNT_0.5 nanocomposite as a function of temperature and applied load.

To investigate the effect of CNTs on the rheological properties of PBS, specifically its flowability, the melt mass-flow rate (MFR) of pure PBS and the PBS/CNT_0.5 nanocomposite was studied at different temperatures and loads (Figure 2). As expected, the MFR of the studied samples increases with rising temperature and applied load. At 180 and 190 °C, the flowability of pure PBS is slightly higher than that of the PBS/CNT_0.5 nanocomposite, regardless of the applied load. However, as the temperature increases to 200 °C, the flowability of the PBS/CNT nanocomposite rises more rapidly than that of pure PBS, eventually surpassing it. It can be assumed that at temperatures above 200 °C, the nanocomposite with 0.5 wt % CNTs is capable of forming an inversion mixture in the melt, leading to a sharp increase in its flowability. It is important to note that the MFR of the PBS/CNT_10 masterbatch could not be determined under the given conditions, as its melt exhibited minimal flow through the plastometer capillary. Therefore, the MFR of the masterbatch was measured at 230 °C and a load of 21.6 kg, yielding approximately 0.04 g/10 min. This indicates that CNTs have a negligible effect on the flowability of PBS at concentrations up to 0.5 wt %; however, when the CNT content increases to 10 wt %, the flowability of PBS significantly decreases (by nearly 10,000 times). Thus, by adjusting the CNT content in PBS, its flowability and processability can be effectively controlled across a wide range.

**Figure 3:**
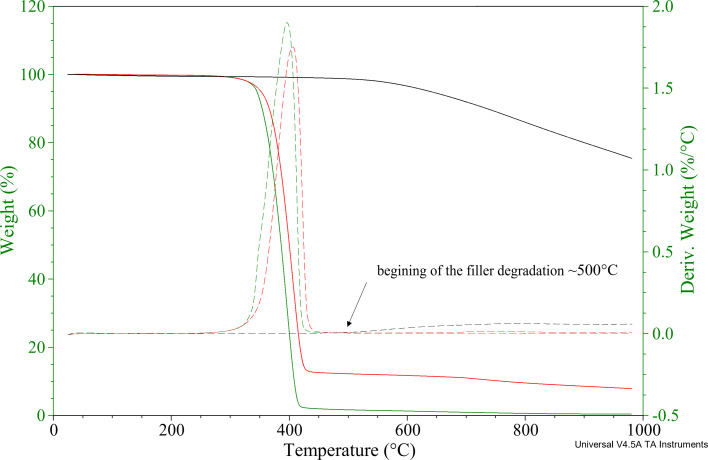
TGA of PBS (green curves), CNTs (black curves), and PBS/CNT_10 masterbatch (red curves).

**Figure 4:**
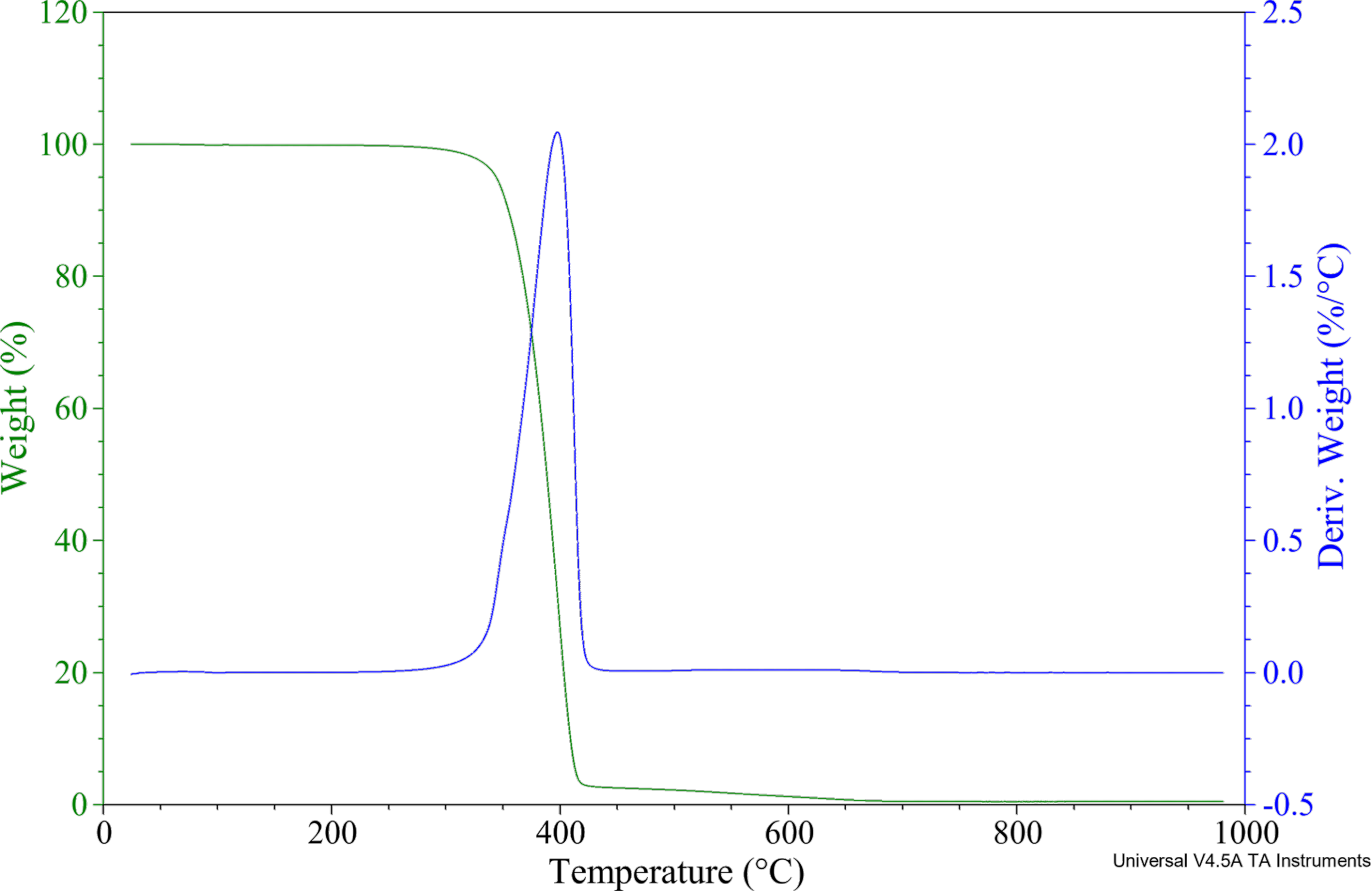
TG (green) and DTG (blue) curves of the PBS/CNT_0.5 nanocomposite.

The thermal stability of the samples and the CNT content in the nanocomposites were determined using thermogravimetric analysis. Figure 3 and Figure 4 present the thermogravimetric (TG) and differential thermogravimetric (DTG) curves of the original PBS, CNTs, and the masterbatch PBS/CNT_10 (Figure 3), as well as of the bio(nano)composite PBS/CNT_0.5 (Figure 4). The numerical results of the TG measurements are provided in Table 1. Based on the TG curve, the *T*_5%_ value was determined, representing the temperature at which 5% of the initial mass of the sample was lost. This *T*_5%_ value was used as a parameter to define the onset of thermal degradation of the material. The residue value (*R*) was also recorded following thermogravimetric analysis. The *T*_max1_ value, indicating the temperature at which the fastest mass loss occurs, was determined from the DTG curve (the first derivative of the TG curve). Figure 3 and Table 1 demonstrate that the masterbatch PBS/CNT_10 exhibits a heat resistance that is 7–8 °C higher than that of pure PBS. The PBS/CNT_0.5 sample shows nearly identical heat resistance (346 °C) to pure PBS, around 345 °C, indicating that such a low concentration of CNTs in PBS has minimal impact on its heat resistance. The thermal resistance of CNTs is 638.9 °C. Thermal degradation of the CNT sample begins at a temperature of 500 °C, with a mass loss of 0.4% at this temperature (Figure 3). Therefore, this temperature was selected for determining the CNT content in the composites. The residual mass of the masterbatch at 500 °C (*R*_500_) is 11.5%, while pure PBS shows a residual mass of 1.8% (Figure 3). Consequently, the CNT content in the sample was calculated to be 10.1% (11.5% + 0.4% − 1.8% = 10.1%). These results confirm the high-quality preparation of the masterbatch with a CNT content of approximately 10 wt %. Following the confirmation of the CNT content in the masterbatch, composites with a CNT content of 0.5 wt % were prepared according to the method described in section “Experimental”. Similarly, the CNT content in the PBS/CNT_0.5 nanocomposite was verified, with TGA data indicating a content of approximately 0.6 wt %.

**Table 1:** Summary of TGA results: degradation temperatures and residue contents of PBS, PBS/CNT composites, and CNTs.

Sample	*T*_5%_ [°C]^a^	*T*_max1_ [°C]^b^	*R*_500_ [%]^c^	*R* [%]^c^	CNT content [%]

PBS	345.0	395.0	1.8	0.5	—
PBS/CNT_10	351.9	406.0	11.5	7.9	≈10.1
PBS/CNT_0.5	346.0	398.0	2.0	0.8	≈0.6
CNTs	638.9	—	99.6	75.4	—

^a^*T*_5%_: temperature at which 5% of the initial sample mass is lost; ^b^*T*_max1_: temperature at which the maximum rate of mass loss occurs, determined from the DTG curve (first derivative of the TG curve); ^c^*R*_500_, *R*: the residue at 500 °C and the final mass residue, respectively.

**Figure 5:**
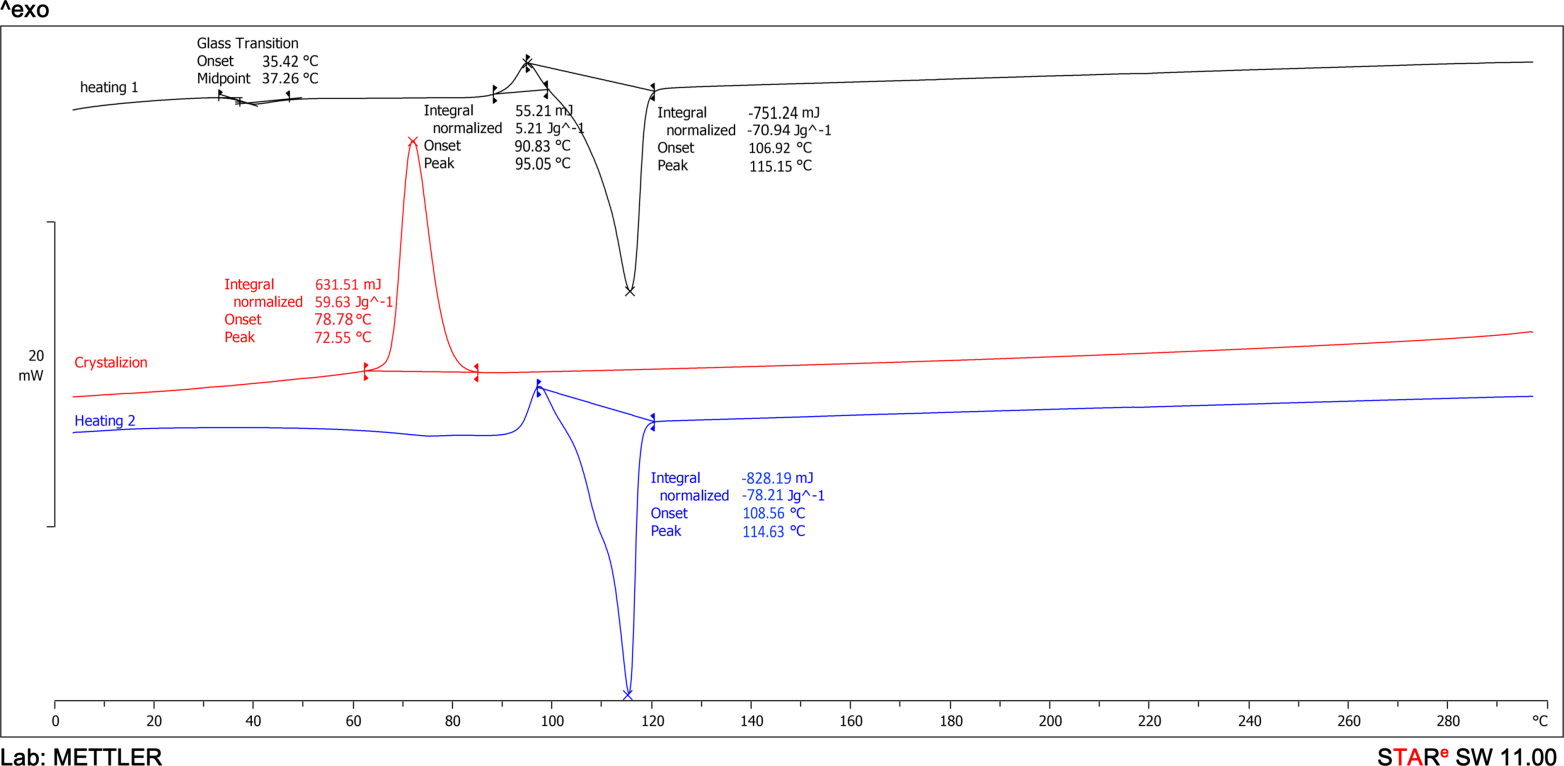
DSC curves of neat PBS.

**Figure 6:**
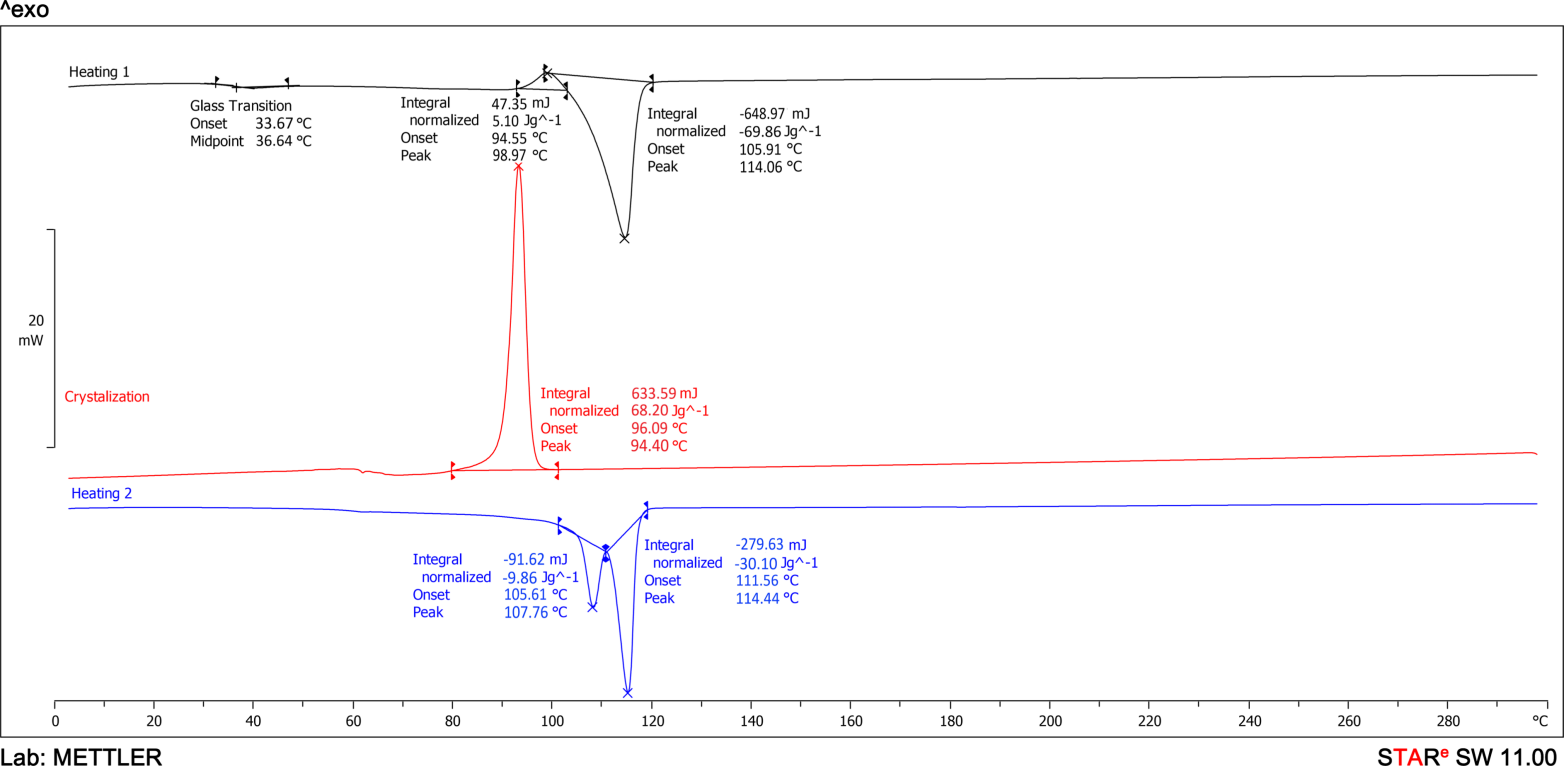
DSC curves of the PBS/CNT_0.5 nanocomposite.

Differential scanning calorimetry (DSC) analysis was conducted to assess the effect of CNTs on the thermal properties and crystallinity of PBS. The DSC thermograms (Figure 5 and Figure 6) allow for the determination of melting temperature (*T*_m_), glass transition temperature (*T*_g_), crystallization temperature (*T*_c_), and enthalpy (Δ*H*) of the studied materials (Table 2). During the first heating cycle, neat PBS exhibited a cold crystallization peak at 95.1 °C and a melting peak at 115.2 °C (Figure 5, black curve). In contrast, the PBS/CNT_0.5 nanocomposite showed a cold crystallization peak at 99.0 °C and a melting peak at 114.1 °C (Figure 6, black curve).

**Table 2:** Thermal transitions of PBS and PBS/CNT_0.5 nanocomposite obtained from DSC analysis.

Sample	Heating 1						Cooling		Heating 2				
	*T*_m_ [°C]^a^	Δ*H*_m_ [J·g^−1^]^b^	*T*_c.c._ [°C]^a^	Δ*H*_c.c_ [J·g^−1^]^b^	*X*_c_ [%]^c^	*T*_g_ [°C]^a^	*T*_c_ [°C]^a^	Δ*H*_c_ [J·g^−1^]^b^	*T*_m_ [°C]^a^	Δ*H*_m_ [J·g^−1^]^b^	*T*_c.c._ [°C]^a^	Δ*H*_c.c_ [J·g^−1^]^b^	*X*_c_ [%]^c^

PBS	115.2	70.9	95.1	5.2	59.6	35.4	72.6	59.6	114.6	78.2	97.5	5.9	65.5
PBS/CNT_0.5	114.1	69.9	99.0	5.1	58.7	33.7	94.4	68.2	107.8 and 114.4	9.9 and 30.1	—	—	9.0 and 27.3

^a^*T*_m_, *T*_c.c._, *T*_g_, *T*_c_: melting temperature, cold crystallization temperature, glass transition temperature, and crystallization temperature, respectively; ^b^Δ*H*_m_, Δ*H*_c.c._, Δ*H*_c_: melting enthalpy, cold crystallization enthalpy, and crystallization enthalpy, respectively; ^c^*X*_c_: degree of crystallinity.

During the cooling process from 300 to 0 °C, crystallization peaks were observed in the thermograms at 72.6 °C for neat PBS (Figure 5, red curve) and 94.4 °C for the PBS/CNT_0.5 nanocomposite (Figure 6, red curve). This indicates that the crystallization temperature of the nanocomposite is nearly 22 °C higher than that of pure PBS, despite the low CNT content.

During the second heating cycle, neat PBS exhibited a cold crystallization peak at 97.5 °C and a melting peak at 114.6 °C (Figure 5, blue curve). In contrast, the PBS/CNT_0.5 nanocomposite sample did not show a cold crystallization peak on the second heating curve, but two melting peaks were observed at 107.8 and 114.4 °C (Figure 6, blue curve). These differences can be attributed to the effect of nanotubes on the PBS structure, leading to the formation of two distinct crystal structures during cooling, which melt at different temperatures (Table 2). The glass transition temperatures of the samples were determined from their first heating curves. The glass transition temperature of PBS is 35.4 °C, while that of the PBS/CNT_0.5 nanocomposite is 33.7 °C.

The PBS sample exhibits a slightly higher degree of crystallinity (Table 2) than the nanocomposite sample during the first heating (59.6% compared to 58.7%), and a significantly higher degree of crystallinity during the second heating (65.5% compared to 27.3% and 9%).

To evaluate the potential of using PBS-based nanocomposites with CNTs, their physical, tribological, and mechanical properties were studied (Table 3 and Table 4). The addition of CNTs to PBS at a concentration of 0.5 wt % has minimal impact on the hardness and water absorption of PBS; however, it significantly influences surface wettability and tribological properties (Table 3). The dynamic and static coefficients of friction for the PBS/CNT_0.5 nanocomposite are 1.7 and 1.3–1.7 times lower, respectively, than those of the pure PBS. The surface wettability of films based on the nanocomposite is 7 mN·m^−1^ higher than that of the original PBS (52 versus 45 mN·m^−1^). This indicates that PBS/CNT_0.5 films will not require additional activation before applying paints, adhesives, or coatings. The addition of CNTs also affects the density of PBS. Specifically, with the incorporation of 0.5 wt % CNTs, the density of PBS increases by 1%, and with the addition of 10 wt % CNTs, the density increases by 5%.

**Table 3:** Tribological and physical properties of PBS and PBS/CNT_0.5 nanocomposite.

Sample	µ_S_^a^		µ_D_^a^		Shore D^b^	*n* [%]^c^	ρ [g·cm^−3^]^d^	σ [mN·m^−1^]^e^
	in	out	in	out				

PBS	0.34 ± 0.03	0.57 ± 0.03	0.27 ± 0.02	0.36 ± 0.03	57 ± 1	0.547 ± 0.013	1.260	≈45
PBS/CNT_0.5	0.26 ± 0.02	0.34 ± 0.03	0.16 ± 0.01	0.22 ± 0.02	58 ± 1	0.557 ± 0.009	1.272	≈52

^a^µ_S_, µ_D_: static and dynamic coefficients of friction, respectively (in: inner side of the film, in contact with the cooling roll; out: outer side of the film); ^b^Shore D: hardness according to Shore D; ^c^*n*: water absorption over 24 h; ^d^ρ: density; ^e^σ: surface tension (wettability).

**Table 4:** Tensile test results for PBS and PBS/CNT_0.5 nanocomposite.

Sample	*R*_m_ [MPa]^a^		δ_m_ [%]^b^		*R*_b_ [MPa]^a^		δ_b_ [%]^b^		*E*_t_ [MPa]^c^	
	MD^d^	TD^e^	MD	TD	MD	TD	MD	TD	MD	TD

PBS	34 ± 0.6	32 ± 0.9	22 ± 1.1	12 ± 1.0	29 ± 0.9	26 ± 2.0	330 ± 23	274 ± 35	480 ± 13	557 ± 31
PBS/CNT_0.5	36 ± 0.7	34 ± 0.7	17 ± 1.7	13 ± 0.5	32 ± 1.0	30 ± 2.0	208 ± 28	125 ± 24	620 ± 22	575 ± 9

^a^*R*_m_, *R*_b_: maximum stress and stress at break, respectively; ^b^δ_m_, δ_b_: strain at maximum stress and the break, respectively; ^c^*E*_t_: tensile modulus; ^d^MD: machine direction; ^e^TD: transverse direction.

The results of tensile tests for PBS and PBS/CNT_0.5 samples are presented in Table 4. The tests were performed in both the machine direction (MD) and transverse direction (TD). The addition of CNTs to PBS at a concentration of 0.5 wt % enhances the mechanical tensile strength and stiffness of the material (tensile modulus increases); however, it significantly reduces its elasticity, as evidenced by a 1.5–2.0 times decrease in relative elongation. The small standard deviations of the measurement results indicate the high homogeneity of the bio(nano)composite.

## Conclusion

A comparison of the results obtained in this study with those reported in the literature reveals a lack of data on the effect of low concentrations (0.5 wt %) of pristine MWCNTs on the structure, crystallization behavior, and rheological, tribological, physical, and mechanical properties of PBS-based nanocomposites prepared via a two-step melt compounding–dilution approach using a masterbatch. While previous studies generally report trends similar to those observed here, such as improved thermal stability, crystallization temperatures, stiffness, and electrical conductivity (not investigated in this work), a significant gap remains regarding tribological performance, surface activity, and rheological responses under varying loads and temperatures.

This study demonstrates that the incorporation of 0.5 wt % MWCNTs into BioPBS significantly modified its properties. The resulting nanocomposite exhibited a 10–15% increase in mechanical strength and a 25–30% improvement in stiffness. Tribological performance was also enhanced, with the coefficient of friction reduced by approximately 40%. Furthermore, the surface wettability increased by 15%, and the crystallization temperature rose by 25%, indicating an acceleration in crystallization kinetics. However, these enhancements were accompanied by a 37–50% reduction in elasticity.

Despite the decrease in ductility, the elongation at break remained at a relatively high level, that is, 208% in the machine direction (MD) and 125% in the transverse direction (TD). Combined with moderate stiffness values (620 and 575 MPa in MD and TD, respectively), these characteristics remain in an acceptable range for certain types of flexible and semi-rigid packaging, which typically require elongation at break in the range of 100–600% and Young’s modulus between 200 and 700 MPa [25]. Potential applications include stretch sleeves, flexible lids, and barrier films, where controlled deformation and improved wear resistance are desired.

Rheological analysis revealed that CNTs decreased the melt flow rate by 5–25% at lower temperatures (180–190 °C), while improving flowability by 5–12% at 200 °C. It is hypothesized that the observed decrease in crystallinity may contribute to enhanced biodegradability. These results suggest promising applications for PBS/MWCNT nanocomposites in semi-rigid packaging and functional components requiring reduced friction. Future research should explore a broader range of CNT concentrations and assess their direct impact on biodegradation behavior.

## Experimental

### Materials

BioPBS™ FZ91PM, purchased from PTT MCC Biochem Company Limited (Bangkok, Thailand), with a density of 1.26 g·cm^−3^, an MFR (190 °C, 2.16 kg) of 5 g/10 min, a melting point of 115 °C, and a puncture impact resistance of 4 kJ·m^−2^, was used.

Industrial-grade multiwalled carbon nanotubes (MWCNTs), purchased from Nanografi NanoTechnology (NG01IM0101), Ankara, Turkey, with a purity of >92%, an outside diameter of 8–28 nm, an inside diameter of 5–10 nm, a length of 10–35 µm, a tap density of 0.15 g·cm^−3^, a true density of 2.2 g·cm^−3^, and a specific surface area of 220 m^2^·g^−1^, were used.

### Preparation of PBS/CNTs nanocomposites

Granulated samples of the PBS nanocomposite were prepared using a co-rotating twin-screw extruder, type BTSK 20/40D (Bühler, Braunschweig, Germany), equipped with screws of 20 mm diameter and an *L*/*D* ratio of 40, and a two-opening die head without degassing [26]. Before processing, PBS was dried in air at a temperature of 80 °C for 5 h. First, a PBS-based masterbatch containing 10 wt % of CNTs (PBS/CNT_10) was obtained. CNTs were manually dosed, and PBS was dosed using a volumetric doser. The temperatures of the individual barrel heating zones were set to 165, 170, 170, and 170 °C. The temperature of the extrusion die-head was set to 175 °C. The screw rotation speed was maintained at constant 150 rpm. The extruded composites were intensively cooled in an air stream and then granulated.

Next, a composite with a CNT content of 0.5 wt % (PBS/CNT_0.5) was produced from the masterbatch using the dilution method. The masterbatch was mixed with the original PBS and dosed into the extruder using a volumetric doser. The temperatures of the individual barrel heating zones were set to 155, 160, 160, and 165 °C. The temperature of the extrusion die-head was set to 165 °C. The screw rotation speed was kept constant at 150 rpm.

The screw was equipped with intensive mixing and kneading elements, including backward elements, to achieve the most uniform distribution and dispersion of CNTs in the polymer matrix [27].

During extrusion, the basic process parameters (Table 5) were recorded: drive torque (*M*), energy consumption (*W*), melt temperature (*T*_d_), and melt pressure (*P*_d_) measured in the die-head.

**Table 5:** Basic extrusion parameters for PBS/CNTs nanocomposites.

Sample	*M* [Nm]^a^	*T*_d_ [°C]^b^	*P*_d_ [bar]^c^	*W* [kW]^d^

PBS/CNT_10	30–32	210–215	25–30	0.60–0.62
PBS/CNT_0.5	19.5–20.1	190–192	13.5–14.5	0.39–0.40

^a^*M*: drive torque; ^b^*T*_d_: melt temperature; ^c^*P*_d_: melt pressure; ^d^*W*: energy consumption.

During the production of the masterbatch PBS/CNT_10, the extruder drive torque reached 32 Nm (Table 5), which is twice as high as that of pure PBS, where the torque value was 14–15 Nm. Additionally, the extrusion of PBS/CNT_10 took place at significantly higher material pressure and temperature, measured in the die, compared to the PBS/CNT_0.5 extrusion. Therefore, the addition of CNTs, at an amount of 10 wt %, significantly complicates the PBS extrusion process due to a sharp increase in melt viscosity. This conclusion is also supported by the results of measuring the melt flow rate of the samples.

**Figure 7:**
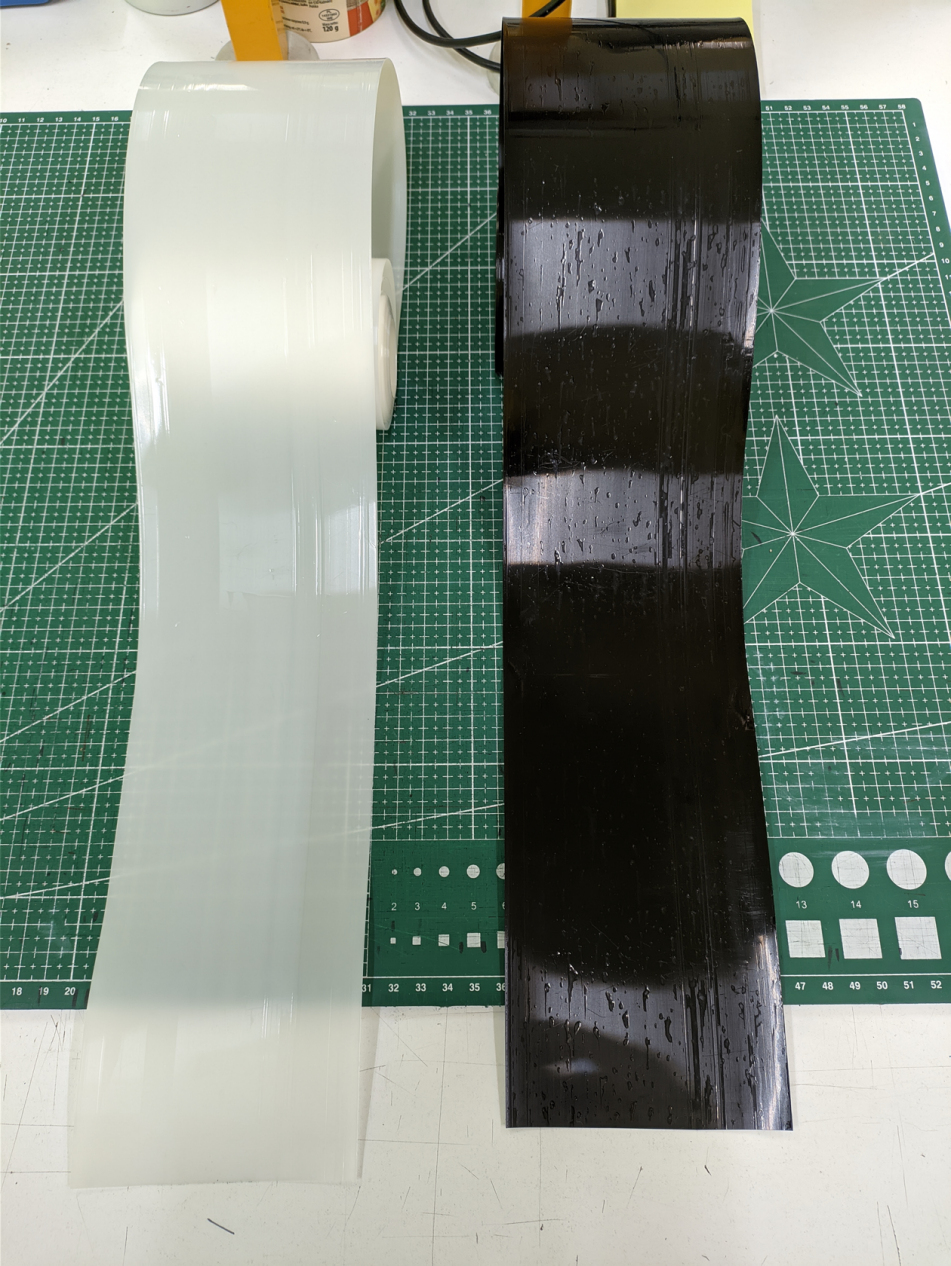
PBS (white) and PBS/CNT_0.5 (black) flat films.

The standardized samples for mechanical, tribological, and physical property testing of the PBS/CNT nanocomposite were obtained from a film (Figure 7) produced using the cast film extrusion method, following a procedure similar to that described in [28]. Barrel zone temperatures of the Plasti-Corder LabStation extruder (Brabender, Duisburg, Germany) were set to 150, 160, and 170 °C, with the die head at 175 °C. The screw speed was 60 rpm, the cooling roll was set to 70 °C, and the rotational speed was 12 min^−1^.

**Figure 8:**
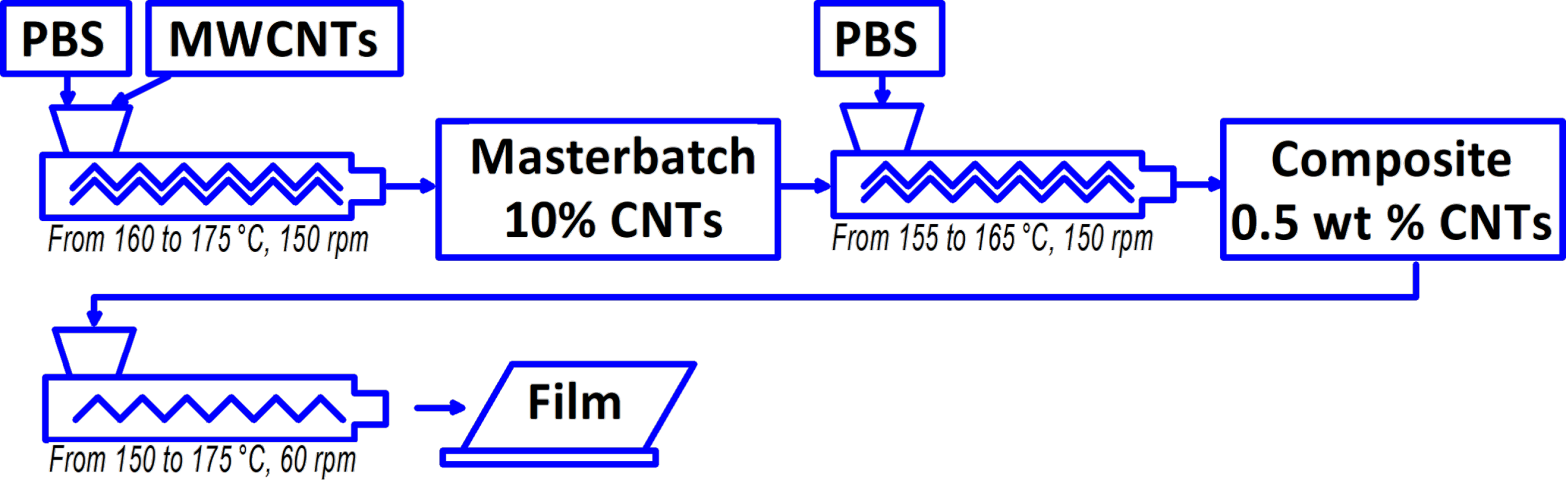
A schematic diagram of the two-stage process for obtaining the nanocomposite and the film based on it.

As a result of the films being freely cast onto the cooling roll, they exhibited different surface layer structures: a smooth surface (marked as “in”) on the side in contact with the cooling roll and a rough surface (marked as “out”) on the opposite side. The flat films obtained had a thickness of approximately 350 μm and a width of about 100 mm. A reference film sample made of original PBS (denoted PBS) was also prepared. A schematic diagram of the two-stage process for obtaining the nanocomposite and the film based on it is shown in Figure 8.

### Characterizations

A scanning electron microscope (SEM), Hitachi SU8010 (Hitachi, Tokyo, Japan), was used to examine the microstructure of the samples and the distribution of the dispersed phase. The microstructure of original CNTs and pellets of PBS and PBS/CNTs nanocomposites was analyzed using the secondary electron (SE) detector at an accelerating voltage of 10 kV. Cross-sectional samples for SEM analysis were prepared using an ion milling system, HITACHI E-3500 (Hitachi, Tokyo, Japan). To enhance conductivity, a 5 nm thick gold layer was sputtered onto all samples using a Cressington 108auto sputter coater (Cressington Scientific Instruments Ltd., Watford, UK). The structural analysis of each material was performed based on a minimum of 20 micrographs for each, taken from multiple samples and various locations within each sample.

A capillary plastometer (Dynisco LMI 4003, Franklin, TN, USA) was used to determine the melt mass-flow rate (MFR) of both original PBS and PBS/CNTs nanocomposites. The MFR was measured following PN-EN ISO 1133-1:2022 “Plastics – Determination of the Melt Mass-Flow Rate (MFR) and Melt Volume-Flow Rate (MVR) of Thermoplastics”. Measurements were conducted at temperatures of 180, 190, and 200 °C, and under loads of 1.2, 2.16, and 21.6 kg to evaluate the influence of temperature and load on the flowability of the composite.

The sample density (ρ) was measured according to PN-EN ISO 1183-3:2003 “Plastics – Methods for Determining the Density of Non-Cellular Plastics – Part 3: Gas Pyknometer Method”, using a helium pycnometer (Anton Paar Ultrapyc 5000, Graz, Austria).

The water absorption (*n*) was measured following ISO 62:2008 “Plastics – Determination of Water Absorption”, using film samples of a square shape (50 × 50 mm^2^) with a thickness of 350 µm.

The surface tension or wettability (σ) of the films was measured according to ISO 8296:2003 “Plastics – Film and Sheeting – Determination of Wetting Tension”. The tests were conducted on the smooth side of the PBS and PBS/CNT_0.5 films.

Dynamic (µ_D_) and static (µ_S_) coefficients of friction tests were conducted using the TIRAtest 27025 testing machine (TIRA Maschinenbau GmbH, Schalkau, Germany), equipped with a specialized attachment for this purpose. Measurements were performed between smooth film surfaces (smooth to smooth) and rough film surfaces (rough to rough). The tests were carried out in accordance with PN-EN ISO 8295:2005 “Plastics – Film and Sheeting – Determination of the Coefficients of Friction” at a measurement speed of 100 mm·min^−1^, using rectangular film samples measuring 80 × 350 mm^2^ with a thickness of 350 µm.

Hardness tests were performed using the Shore method in accordance with ISO 868:2003 “Plastics and Ebonite – Determination of Indentation Hardness by Means of a Durometer (Shore Hardness)”. Square film samples (50 × 50 mm^2^) with a thickness of 350 µm were stacked until the required thickness of 4 mm was achieved. Shore D hand-held hardness testers (Zwick Roell Group, Ulm, Germany), along with a specialized test stand with a load weight (Zwick Roell Group, Ulm, Germany), were used to ensure precise positioning of the hardness tester at a right angle to the specimen surface.

Mechanical properties tests under static tension were conducted using a TIRAtest 27025 testing machine (TIRA Maschinenbau GmbH, Schalkau, Germany). Maximum stress (*R*_m_), stress at break (*R*_b_), tensile modulus (*E*_t_), strain at maximum stress (δ_m_), and strain at break (δ_b_) were determined according to PN-EN ISO 527-3:2019-01 “Plastics – Determination of Tensile Properties – Part 3: Test Conditions for Films and Plates”. The tests were conducted at an extension rate of 1 mm·min^−1^ for tensile modulus measurements and 100 mm·min^−1^ for other parameters, in both MD and TD, using rectangular film samples measuring 15 × 150 mm^2^.

A thermogravimetric analyzer (TGA), Q500 (TA Instruments, New Castle, DE, USA), was used to determine the thermal stability and CNT content, following a procedure similar to [29]. Prior to testing, all samples were dried at 70 °C for 8 h in an air dryer.

Thermal studies were conducted using a differential scanning calorimeter (DSC), type DSC 1 STARe System (Mettler Toledo, Columbus, OH, USA), following a procedure similar to [29–30]. The thermal cycle included heating from 0 to 300 °C at a rate of 10 °C·min^−1^, annealing at 300 °C for 3 min, cooling to 0 °C at 10 °C·min^−1^, and reheating to 300 °C at 10 °C·min^−1^. The Universal STARe SW 11.00 software (Mettler Toledo) was used to analyze the obtained data and calculate the degree of crystallinity of PBS, assuming a thermodynamic fusion enthalpy of 110.3 J·g^−1^ for 100% crystalline PBS [31].

## Data Availability

Data generated and analyzed during this study is available from the corresponding author upon reasonable request.
